# Recent Developments in Ring-Opening Copolymerization of Epoxides With CO_2_ and Cyclic Anhydrides for Biomedical Applications

**DOI:** 10.3389/fchem.2021.647245

**Published:** 2021-04-20

**Authors:** Xue Liang, Fei Tan, Yunqing Zhu

**Affiliations:** ^1^School of Materials Science and Engineering, Tongji University, Shanghai, China; ^2^Department of Orthopedics, Shanghai Tenth People's Hospital, Tongji University School of Medicine, Shanghai, China; ^3^Department of Otorhinolaryngology-Head and Neck Surgery, Shanghai East Hospital, Shanghai, China; ^4^School of Medicine, Tongji University, Shanghai, China

**Keywords:** degradable polymers, Lewis pairs, biomedical applications, post-polymerization modifications, ring-opening copolymerization

## Abstract

The biomedical applications of polyesters and polycarbonates are of interest due to their potential biocompatibility and biodegradability. Confined by the narrow scope of monomers and the lack of controlled polymerization routes, the biomedical-related applications of polyesters and polycarbonates remain challenging. To address this challenge, ring-opening copolymerization (ROCOP) has been exploited to prepare new alternating polyesters and polycarbonates, which would be hard to synthesize using other controlled polymerization methods. This review highlights recent advances in catalyst development, including the emerging dinuclear organometallic complexes and metal-free Lewis pair systems. The post-polymerization modification methods involved in tailoring the biomedical functions of resultant polyesters and polycarbonates are summarized. Pioneering attempts for the biomedical applications of ROCOP polyesters and polycarbonates are presented, and the future opportunities and challenges are also highlighted.

## Introduction

The polymerization of carbon dioxide (CO_2_) with epoxides to form polycarbonate can maximize the use of CO_2_ to achieve value-adding benefits, in which polycarbonate has great potential for biomedical applications due to its excellent optical and mechanical properties, as well as good biocompatibility (Paul et al., 2015b; Cui et al., 2019; Ye et al., 2019). Compared with petroleum derivative monomers, bio-based monomers usually have more functional groups, and it is difficult to achieve highly selective polymerization through traditional polymerization methods (Stadler et al., 2019). For ring-opening copolymerization (ROCOP) of epoxide with CO_2_ or cyclic anhydrides, many efficient metal-based catalysts have been developed to produce alternating polycarbonates or polyesters (Paul et al., 2015b; Longo et al., 2016; Xia and Zhao, 2018; Liang et al., 2021), including zinc (Deacy et al., 2018, 2020a; Denk et al., 2020), cobalt (Li et al., 2017a; Ambrose et al., 2019), aluminum (Sanford et al., 2018), or nickel (Huang et al., 2017; Li et al., 2017a) complexes with different ligands. These organometallic catalysts exhibit excellent catalytic performance and high selectivity toward the formation of ester or carbonate linkages during polymerizations, showing reduced side reactions forming ether linkages or cyclic carbonates. In recent years, dinuclear metal catalysts have received widespread attention due to their higher catalytic efficiency than mononuclear catalysts (Yu et al., 2016; Garden et al., 2017), due to the synergy between metals (Cheng et al., 2019). Besides, many studies have shown that dinuclear catalysts usually showed an excellent catalytic activity even in the absence of cocatalysts (Kember and Williams, 2012; Saini et al., 2014). Dinuclear metal catalysts generally exhibited a higher catalytic activity and selectivities as well as fewer side reactions, which could be advantageous for making biopolymers with well-defined chain structures.

Despite the fact that metal-based catalysts have shown excellent catalytic performance, there are also some problems such as complicated ligand design and synthesis procedure in order to reach high catalytic ability (Saini et al., 2014). In addition, the removal of residual metal substances in the system after the polymerization is also a problem that needs attention (Alferov et al., 2019). Residual trace of metal from organometallic catalysts may pose potential toxicity problems that limit their applications in biomedicine (Nachtergael et al., 2015). In order to overcome this issue, organic/metal-free catalysts with efficiency and selectivity/control have been extensively studied in the last decade (Lin et al., 2019; McGraw and Chen, 2020; Hong, 2021). The development of metal-free Lewis pair catalyst systems are considered an effective way. Li's team has developed a series of Lewis pair systems that display good catalytic ability and selectivity (Ji et al., 2018a,b; Ji et al., 2019; Wang et al., 2020). Li et al. successfully copolymerized phthalate with propylene/ethylene oxide at room temperature using a two-part catalyst containing phosphazene base and triethyl borane (TEB) to produce block copolymers with strict AB-type sequence structure and controlled molecular weights (Li et al., 2019a). Zhang et al. synthesized alternating copolymers with high molecular weights (up to 34 kg·mol^−1^) with metal-free Lewis pairs [phosphazene base (*t*-BuP_1_) as the base and TEB as the acid; Zhang et al., 2020a]. Furthermore, no metal substance being introduced during the polymerization process could be an advantage to expand its application in biomedicine.

One of the limitations of ROCOP is that some functional groups are incompatible during the polymerization process, such as hydroxyl, primary or secondary amines, carboxylic acid, or other protonic functional groups, which could function as chain transfer agents, thereby affecting the molecular weight and distribution of the polymer. Besides, the coexistence of species with different degrees of functionality usually leads to broad or even multimodal molecular weight distributions, which also jeopardizes the chain-end fidelity. Moreover, excessive protonic functional groups may cause catalyst deactivation (Yi et al., 2019). Therefore, post-polymerization modification is an important means to realize multi-functionalities on ROCOP polymers, and the desired functional groups can be accurately connected to the polymer chain by different modification methods (Ntoukam et al., 2020). In addition, the functionalization of the polymer can be achieved without protecting and deprotecting processes for functional monomers. For instance, Sanford et al. incorporated both aldehyde and alkene functionalities into the polymer to achieve orthogonal post-polymerization modification in a one-pot process (Sanford et al., 2018).

**Figure 1:**
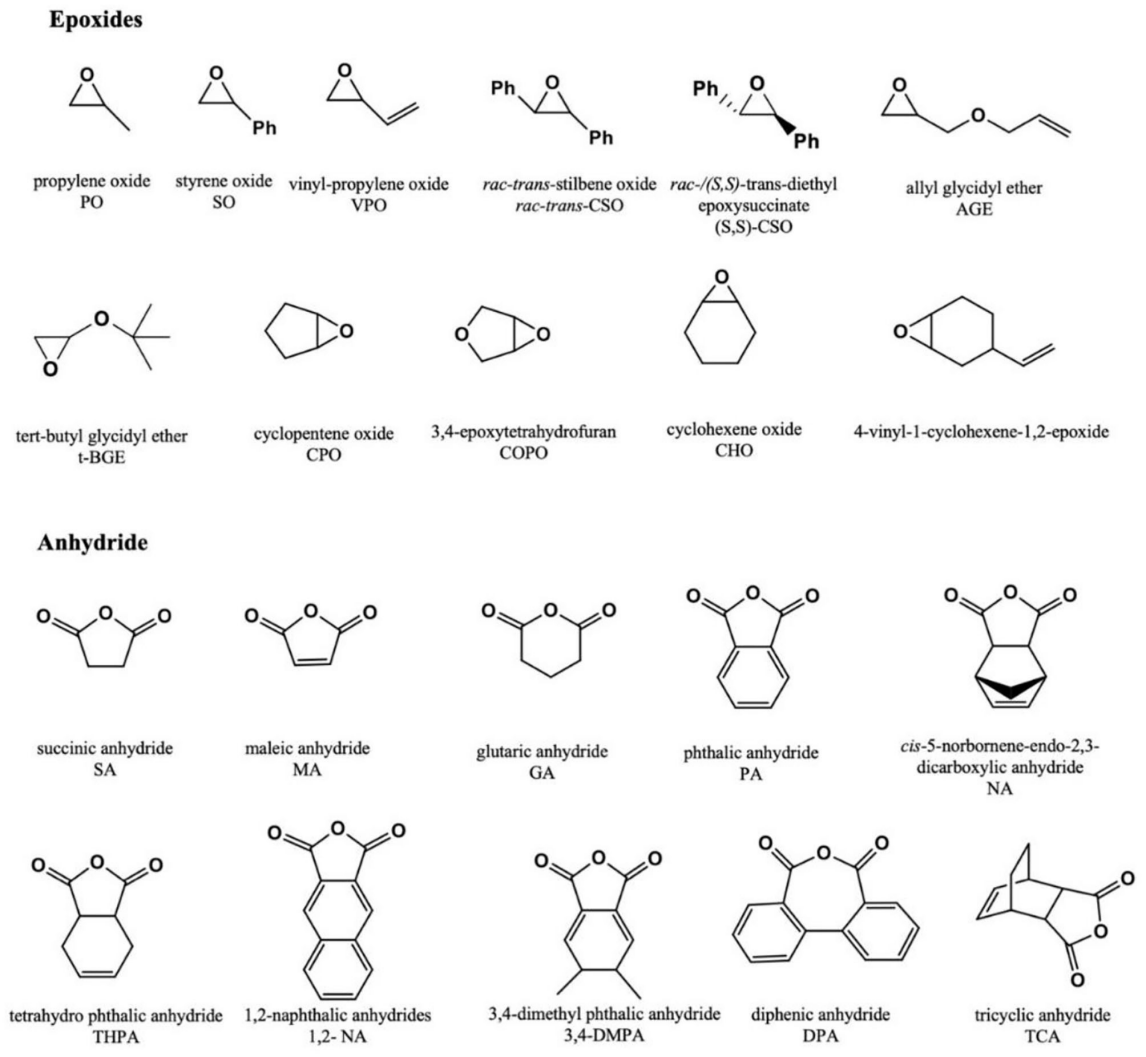
The structures of epoxides and anhydrides discussed in this review.

**Scheme 1:**
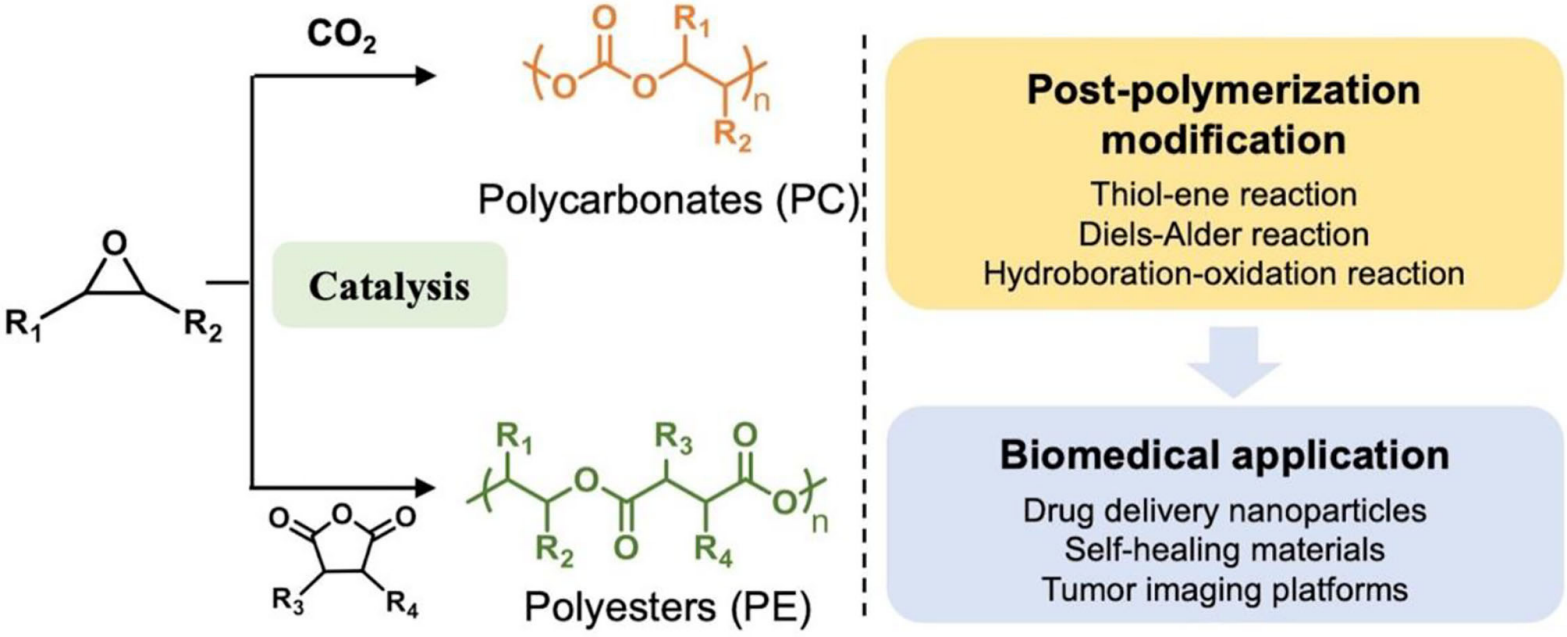
Ring-opening copolymerization of epoxides with CO_2_ and cyclic anhydrides and post-polymerization modification for biomedical applications.

Regarding the advantages of dinuclear complexes and metal-free Lewis pairs for making polymers with potential applications in biomedicine, this review focuses on the recent development of ROCOP of epoxides/CO_2_ or cyclic anhydrides (Figure 1) to produce polycarbonates, polyesters, or copolymers, with novel homo- and heterodinuclear metal catalysts and metal-free Lewis pair catalyst systems. Furthermore, the post-polymerization functionalization of polycarbonates or polyesters will be summarized, and the influence that multifunctionalities of polymer exert over potential biomedical applications will be emphasized (Scheme 1).

## Catalyst Systems

### Homodinuclear Catalysts

**Figure 2:**
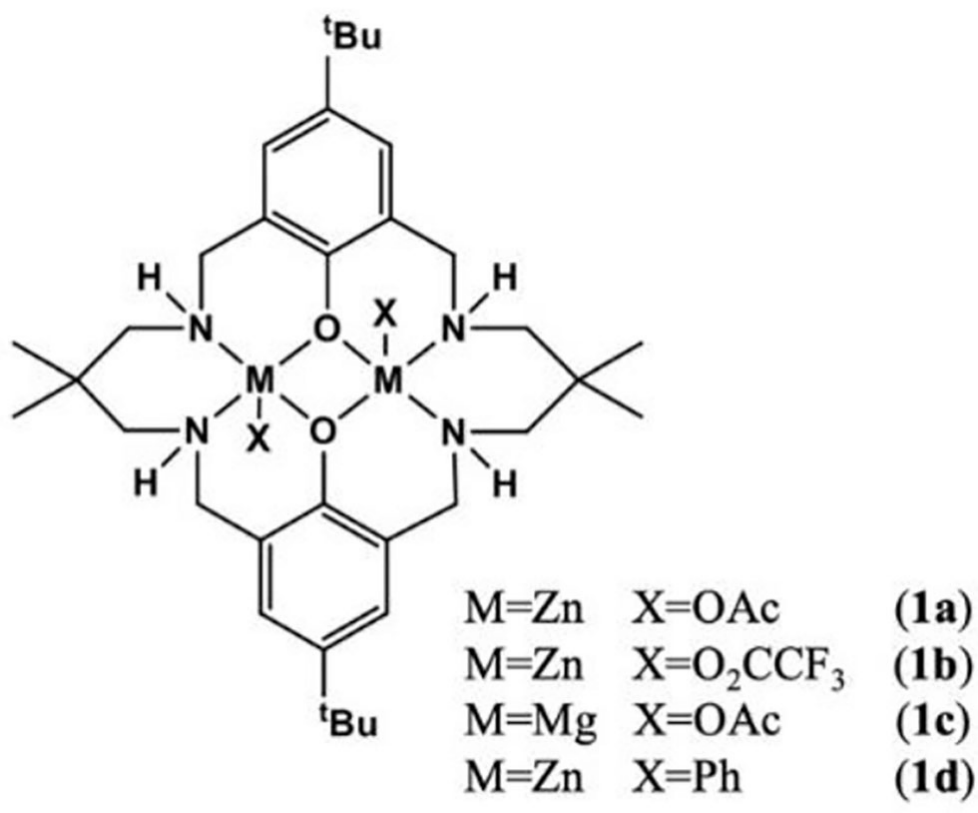
The structures of various homodinuclear catalysts.

For the ROCOP, many types of mononuclear or dinuclear organometallic catalysts have been developed (Paul et al., 2015b; Longo et al., 2016). In order to achieve better catalytic efficiency, cocatalysts are usually required for mononuclear complexes (Paul et al., 2015b; Thevenon et al., 2018). However, for dinuclear complexes, the addition of cocatalysts can be avoided. Besides, bimetallic catalysts show good catalytic performance due to the synergy between the metal centers (Zhou et al., 2017). The Williams group has prepared a series of dizinc catalysts, including LZn_2_(X)_2_ [X = OAc, O_2_CCF_3_, phenyl (Ph)] (Romain and Williams, 2014; Thevenon et al., 2015; Yi et al., 2015; Romain et al., 2017) coordinated by a macrocyclic ligand (Figure 2), which exhibits excellent catalytic efficiency and high selectivity for ROCOP of CO_2_ and epoxides at relatively low CO_2_ pressure (~1 bar). In 2015, they used the waste CO_2_ to carry out a copolymerization experiment with cyclohexene oxide (CHO), using LZn_2_(OAc)_2_, LMg_2_(OAc)_2_, and LZn_2_(O_2_CCF_3_) as the catalysts. These catalysts showed good tolerance, even if the CO_2_ was contaminated by compounds containing S–H (H_2_S, octadecanethiol), N–H [diethylamine, monoethanolamine (MEA)], and O-H (H_2_O, MEA, SO_2_) groups (Chapman et al., 2015).

Multiblock copolymers usually consist of different polymer blocks that are covalently bonded to each other (Yoshida and Takenaka, 2015), which combines different properties exhibited by each block to improve the performance and functionality. One-pot synthesis could be an effective method to develop block polymers, and the key is to control the growth sequence of polymer chains to obtain predictable and well-defined copolymers. In 2008, Coates and co-works used (bdi)ZnOAc complex to catalyze the one-pot terpolymerization of epoxide, anhydride, and CO_2_ to afford block copolymers. Kinetic studies suggested a highly selective product-determining step (Jeske et al., 2008). In 2014, a new type of chemoselective control has been proposed by Williams. In the process of ring-opening polymerization (ROP) and ROCOP, it can selectively synthesize polyesters and polycarbonates (Romain and Williams, 2014; Paul et al., 2015a).

**Figure 3:**
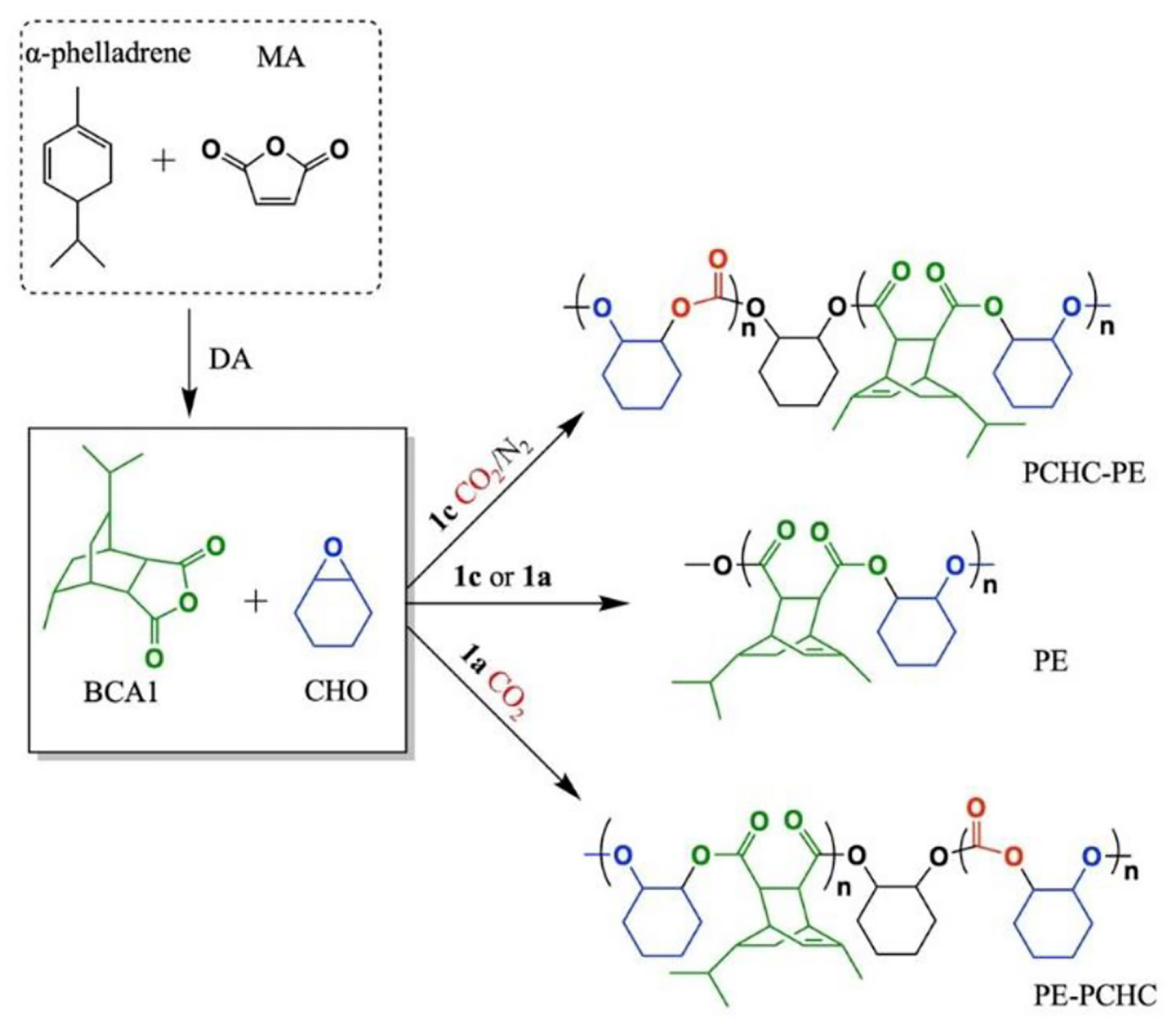
Polyesters and block copolymers based on the bio-derived anhydride, BCA1, were produced using catalysts LZn_2_(OAc)_2_ (**1a**) and LMg_2_(OAc)_2_ (**1c**). The images are reproduced from Saini et al. (2017).

In previous studies on chemoselective polymerization, both AB and ABA type block were formed, resulting in bimodal molecular weight distributions of the copolymer (Romain and Williams, 2014). In order to avoid this issue, the LZn_2_Ph_2_ catalyst was proposed to catalyze ROCOP of CHO/phthalic anhydride (PA), in the presence of cyclohexane diol as the initiator. As the phenyl co-ligand was hydrolyzed by cyclohexane diol during initiation, the obtained poly(cyclohexylene phthalate) (PCHPE) featured a dihydroxyl telechelic structure. In the terpolymerization, the ROCOP of CHO/PA occurred before the ROP of ε-decalactone (ε-DL) to form pure PCHPE. After PA was completely consumed, the ROP of ε-DL was activated. Given the fact that first formed PCHPE featured a dihydroxyl telechelic structure, ABA type block (PDL-*b*-PCHPE-*b*-PDL) was formed at the end of polymerization (Zhu et al., 2015). Then, the chemoselective concept was extended to the tetrapolymerization of epoxide, lactone, cyclic anhydride, and CO_2_, demonstrating that LZn_2_(OAc)_2_ catalysts can switch between different polymerization cycles and exhibit high monomer selectivity (Romain et al., 2016). In particular, LZn_2_(OAc)_2_ and LMg_2_(OAc)_2_ catalysts showed opposite selectivity in the ROCOP of CHO/BCA1/CO_2_ to prepared copolymers, where BCA1 is a tricyclic bio-derived anhydride usually synthesized *via* Diels–Alder reaction between α-phellandrene and maleic anhydride (MA) (Figure 3). When LZn_2_(OAc)_2_ was added to reaction, the ROCOP of CHO and BCA1 occurred first. Then after the complete consumption of BCA1, the ROCOP of CHO/CO_2_ started to initiate, leading to the formation of poly(ester-*b*-carbonate). Contrarily, when LMg_2_(OAc)_2_ was used, the ROCOP of CHO and CO_2_ occurred first, and only upon the removal of CO_2_ was the ROCOP of CHO/BCA1 able to initiate to afford poly(carbonate-*b*-ester) (Saini et al., 2017). This opposite selectivity is due to the fact that LZn_2_(OAc)_2_ and LMg_2_(OAc)_2_ catalysts have different reaction orders in the anhydride.

**Figure 4:**
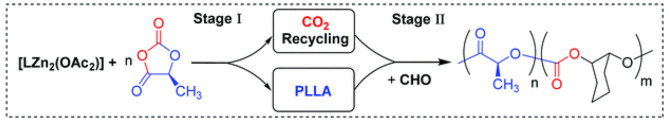
Proposed polymerization pathway whereby the di-zinc catalyst bridges two catalytic cycles: LLA-OCA ROP and CO_2_/CHO ROCOP. LLA-OCA, l-lactide-*O*-carboxyanhydride; ROP, ring-opening polymerization; ROCOP, ring-opening copolymerization. Adapted with permission from Raman et al. (2019). Copyright 2019 The Royal Society of Chemistry.

Recently, chemoselective polymerization was further extended to the terpolymerization of *O*-carboxyanhydride, CHO, and CO_2_, to prepare PLA-*b*-PCHC. This strategy is different from that of the previous studies, making PLA-*b*-PCHC by sequential addition method or tandem catalysis. Instead, two different polymerization cycles are coupled together by a one-pot synthetic strategy with LZn_2_(OAc)_2_ as the only catalyst. The PLLA was prepared by the ROP of l-lactide-*O*-carboxyanhydride (LLA-OCA), and then the CO_2_, released by OCA during its ROP, was cooperated with CHO to synthesize PCHC blocks by ROCOP (Figure 4). In this reaction, ROCOP happened after the ROP of LLA-OCA, and the IR signals representing PCHC increase after the LLA-OCA was completely consumed. Up to 91% of the CO_2_ was incorporated into block copolymers, with only 9% forming cyclic carbonate due to the backbiting of the polymer chain (Raman et al., 2019).

**Figure 5:**
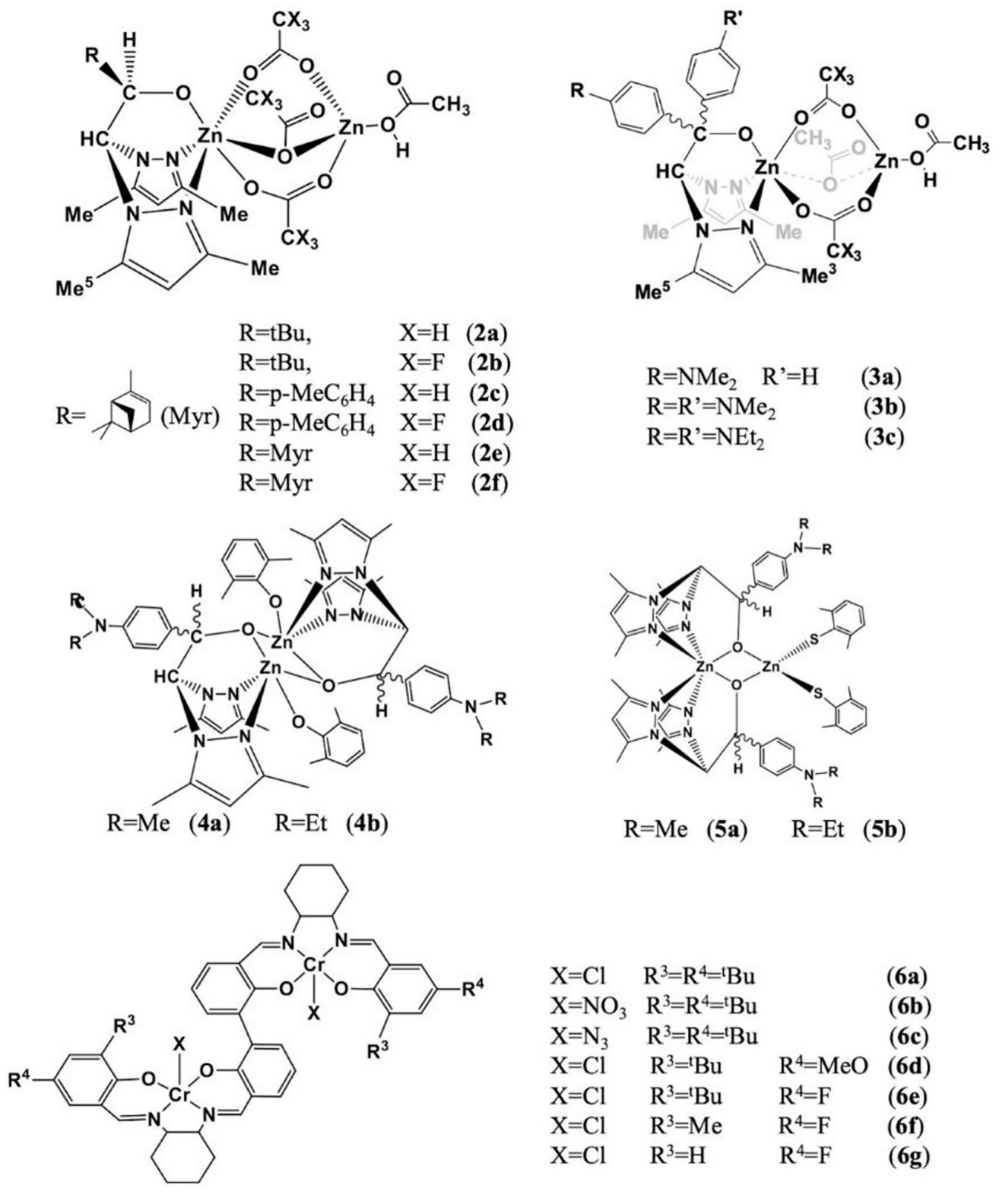
The structures of the catalysts **2–6** based on scorpionate ligand.

Lara-Sanchez and co-workers prepared a series of dinuclear zinc catalysts using scorpionate ligands (Figure 5) for the ROCOP of epoxides and CO_2_ to form random polycarbonates. In catalysts **2** (Martínez et al., 2016) and **3** (de la Cruz-Martinez et al., 2020), the heteroscorpionate ligand is coordinated to a zinc center by κ^3^-NNO coordination mode, three acetate co-ligands bridging the 2 six- and four-coordination zinc centers. The ROCOP of CO_2_ and CHO was carried out under solvent-free, cocatalyst-free, optimal reaction temperature, and CO_2_ pressure. It was found that all catalysts can initiate copolymerization without the presence of co-catalysts. Among them, catalyst **2e** showed the highest catalytic activity [89% conversion, turnover frequency (TOF) up to 5.56 h^−1^], carbonate linkage content (99%), and polycarbonate selectivity (90%). After that, in 2020, another type of dinuclear zinc catalysts based on chiral/thioalkoxide NNO-scorpionate ligand was synthesized (Sobrino et al., 2020). The difference from the previous study is that in catalysts **4–5**, the heteroscorpionate ligand is connected to the zinc center through two nitrogen atoms on the pyrazole rings, and the oxygen atom on the alkoxy group is connected to the two zinc by κ^2^-NNμ-O coordination mode. In addition, each zinc center is coordinated with an aryloxy ligand (Figure 5, cat. 4–5). In the ROCOP of CHO and CO_2_, in the absence of a co-catalyst, catalyst **4** shows the best catalytic activity and carbonate selectivity [75% of conversion, carbonate bond content up to 99%, and high PCHC/cyclohexene carbonate (CHC) ratio 93/7].

Lu and coworkers prepared a series of dinuclear chromium complexes based on bisphenol linking bridge (Figure 5, cat. 5), which can be used for the ROCOP of epoxides [propylene oxide (PO), phenyl glycidyl ether (GO), styrene oxide (SO), 4-chlorostyrene oxide (Cl-SO), epichlorohydrin (ECH), and various meso-epoxides: cyclopentene oxide (CPO), 3,4-epoxytetrahydrofuran (COPO), 1,2-epoxy-4-cyclohexene (CEO), *cis*-2,3-epoxybutane (CBO), and 1,4-dihydro-naphthalene oxide (CDO)] with cyclic anhydrides [SA, glutaric anhydride (GA), diglycolic anhydride (DGA), PA, and MA] or CO_2_ or dihydrocoumarin (DHC), with co-catalysts PPN-X (X = Cl, NO_3_, N_3_). Besides, these catalyst systems can also be used for the one-pot alternating terpolymerization of PO/PA/CO_2_, PO/PA/DHC, or PO/CO_2_/DHC to prepare polyester-*b*-polycarbonate, polyester-*b*-polyester, or poly(ester-*co*-carbonate) (Liu et al., 2018). Later, this group developed polyesters with completely alternating structures based on the similar catalyst system and trans-internal epoxides [*trans*-CSO, (*S*,*S*)-CSO, and *trans*-CTO] and cyclic anhydrides (PA, 1,2-NA, 3,4-DMPA, DPA, and GA). All polyesters were stereoirregular, where poly(*rac*-*trans*-CSO-*alt*-1,2-NA) and poly(*rac*-*trans*-CSO-*alt*-3,4-DMPA) were amorphous with high *T*_g_ values of 197 and 178°C, respectively, but the poly[(*S*,*S*)-CSO-*alt*-1,2-NA] and poly[(*S*,*S*)-CSO-*alt*-3,4-DMPA] were semi-crystalline polymers with melting temperatures (*T*_m_) of 256 and 280°C, respectively. The poly[(S,S)-CSO/DPA], poly[(S,S)-CSO/GA], and poly[(S,S)-CTO/PA] were stereoirregular and amorphous. This study also pointed out that the substituents of the cyclic anhydride backbone and the aromatic structure with higher rigidity can significantly improve crystallinity and thermostability (Liu et al., 2019).

**Figure 6:**
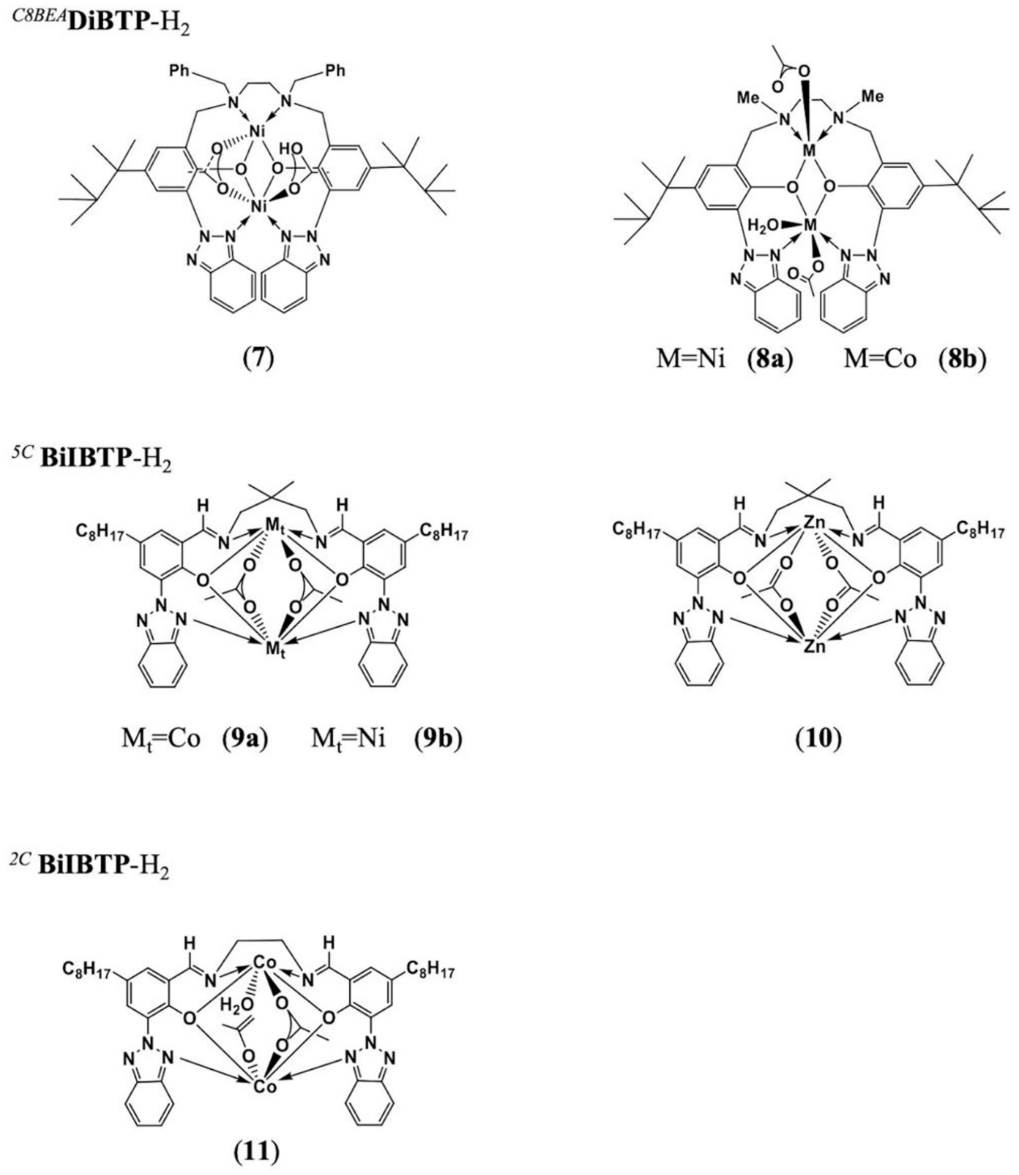
The structures of catalysts **7–8** or **9–11** based on DiBTP-H_2_ or BiIBTP-H_2_, respectively.

The Ko group has proposed a series of multinuclear metal catalysts, for instance, di-nickel (Lin et al., 2016; Tsai et al., 2016; Huang et al., 2017; Li et al., 2019b; Su et al., 2020a,b), di-zinc (Chang et al., 2016), di-cobalt (Su et al., 2019b), and bi-yttrium (Su et al., 2019a), using amine bis(benzotriazole phenolate) and its derivatives as the ligand, for the ROCOP of epoxides with CO_2_ or cyclic anhydrides (Su et al., 2014; Chuang and Ko, 2015; Yu et al., 2016; Li et al., 2017a; Chang et al., 2018). In 2016, the Ko group reported the heat-resistant metal catalysts, bearing multidentate diamine-bis(benzotriazole phenolate) (DiBTP) derivatives (Figure 6, cat. 7–8), which remained active at a temperature of up to 140°C. **8a** showed a better catalytic activity than **7** due to the less sterically encumbered coordination sphere, which promotes the coordination of CHO monomers or the insertion of CO_2_ during the copolymerization. **8a** can also catalyze the copolymerization of VCHO with CO_2_ to produce cross-linkable poly(vinyl cyclohexene carbonate) (Lin et al., 2016). Then this group also synthesized a series of cobalt, nickel, and zinc complexes based on bis(benzotriazolyliminophenolate) (BilBTP) derivatives (Figure 6, cat. 9–11; Yu et al., 2016).

### Heterodinuclear Complex Catalyst

With the development of bimetallic catalysts for ROCOP of epoxides/cyclic anhydrides or CO_2_, homodinuclear catalysts can produce completely alternating copolymers, but the small scope of ligands and metals limits its development (Trott et al., 2019). Over the last decade, a series of heterodinuclear metallic catalysts have emerged with excellent catalytic performance due to the synergetic effect between metal centers. Detailed kinetic, mechanistic, and theoretical studies suggest a “chain shuttling” mechanism where the rate-limiting step is metal–carbonate attack on a coordinated epoxide; thus, shuttle and activation of monomers at different metal centers can improve catalyst performance (Trott et al., 2019; Deacy et al., 2020a). Moreover, these studies demonstrated that heterodinuclear metal complexes may have better thermodynamic stability than the homodinuclear analogs (Deacy et al., 2018; Trott et al., 2019). This section mainly summarizes the latest progress in the development of heterodinuclear catalysts and their catalytic activity for ROCOP of epoxides and cyclic anhydrides or CO_2_.

In 2014, the Williams group reported a heterodinuclear Zn(II)/Mg(II) complex based on the macrocyclic ligand (L). For the ROCOP of CO_2_ and epoxides, the heterodinuclear catalyst showed a much higher catalytic activity (TOF = 79 h^−1^) than LZn_2_(OAc)_2_ (TOF = 17 h^−1^) or LMg_2_(OAc)_2_ (TOF = 52 h^−1^) or their blends [molar ratio of LZn_2_(OAc)_2_:LMg_2_(OAc)_2_ = 1:1, TOF = 40 h^−1^]. But it is difficult to separate the target product during the preparation of the catalyst (Saini et al., 2014). To address the purification problem, sequential metalation reaction method was used, in which the monometalization of the ligand was first achieved, followed by the addition of the second metal center. In the ROCOP of CHO with CO_2_ or PA, the pure LMgZnBr_2_ (**12l**) showed the highest catalytic efficiency, compared with the homodinuclear complexes or their mixtures, suggesting a synergetic effect between different metals (Garden et al., 2015).

**Figure 7:**
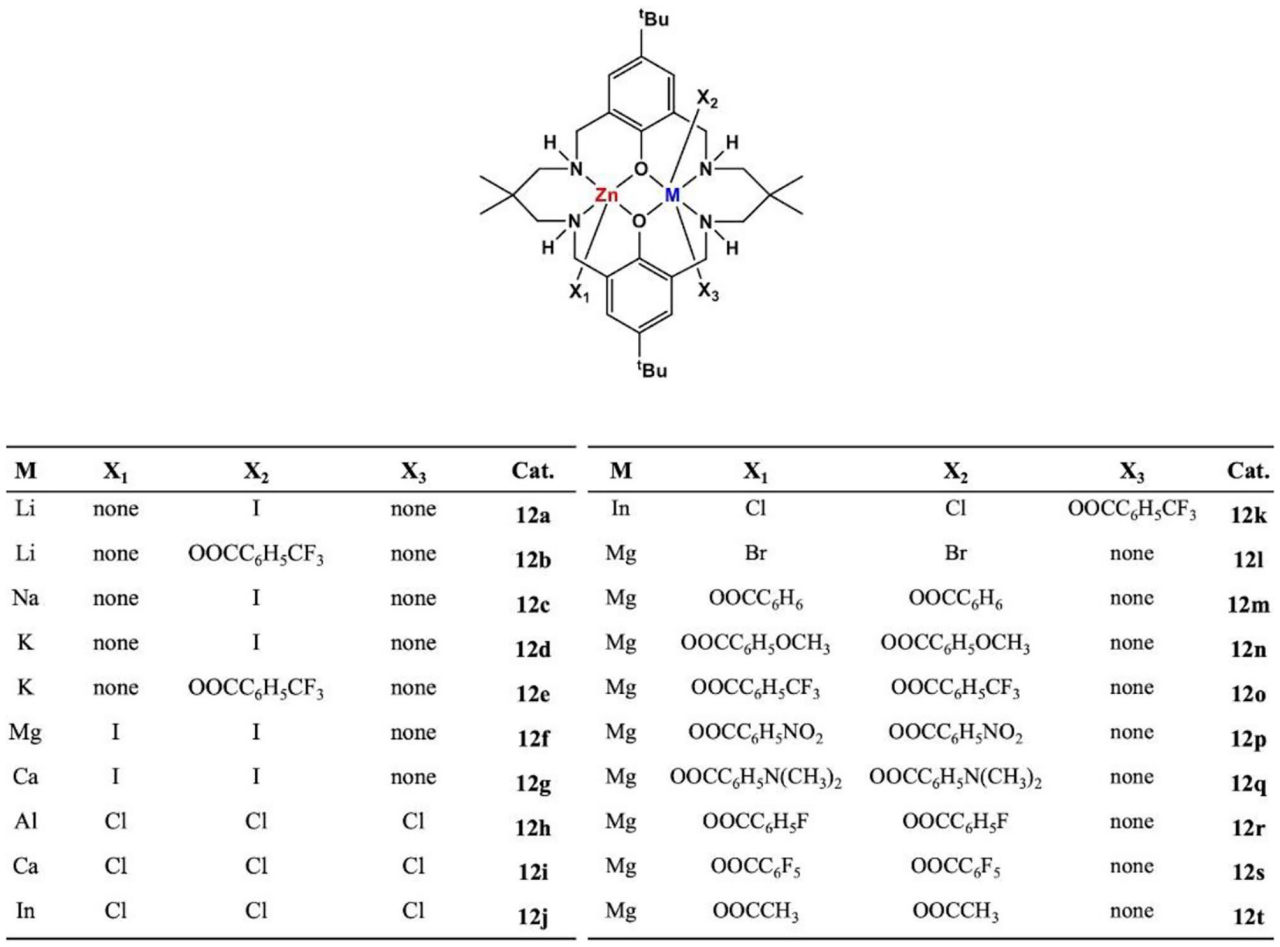
The structures of a series of hetero-Zn/M catalysts based on macrocyclic ligand.

The type of metal center can significantly affect the polymerization rate, chemoselectivity, molar mass, and structure of the ROCOP polymers. Since the metals from Group 1 or 2 are highly Lewis acidic, considering this feature may enhance the coordination of epoxides, and it is possible to generate unstable metal–carbonate bonds to lead a faster catalytic rate. A series of heterodinuclear combinations, featuring Zn(II)/M (Deacy et al., 2018, 2020a), were chelated by the macrocyclic ligand (Figure 7). For ROCOP of CHO/CO_2_, the most active and selective catalyst feature was Mg(II)/Zn(II), while all other heterodinuclear combinations showed a weaker activity.

In 2019, eight new heterodinuclear metal complexes featuring carboxylate co-ligands were developed, and their catalytic ability for the ROCOP of CHO/CO_2_ was investigated (Figure 7, **12m−12t**; Trott et al., 2019). Under 1-bar CO_2_ pressure and 0.1 mol% catalyst loading, all the heterodinuclear metal complexes were active for the ROCOP at 80°C, producing polycarbonates with high carbonate linkage content and exhibited up to 99% CO_2_ absorption. Among these complexes, Zn(II)/Mg(II) complex demonstrated the highest catalytic efficiency (TOF = 124 h^−1^), which is significantly higher than the di-Zn(II) (TOF = 18 h^−1^) and di-Mg(II) (TOF = 30 h^−1^) complexes. Kinetic studies have shown that reaction has a first-order dependence on catalyst and epoxide concentration but a zero-order dependence on CO_2_ pressure (10–40 bar).

To further investigate the synergetic effect, the difference in catalytic activities between the MgCo heterodinuclear catalysts and the MgMg and CoCo homodinuclear catalysts was investigated. The synergetic effect was most likely due to the fact that the magnesium center enhances the transition state entropy by reducing the barriers of epoxide coordination, while the cobalt center reduces the transition state enthalpy by enhancing the nucleophilicity of the cobalt carbonate. This study highlights the potential for heterodinuclear synergy and emphasizes the importance of metal selection based on the specific role of metals in the cycle. Further applications of this mechanism can be made with other homodinuclear and heterodinuclear catalysts (Deacy et al., 2020b).

### Metal-Free Lewis Pair Catalyst System

**Figure 8:**
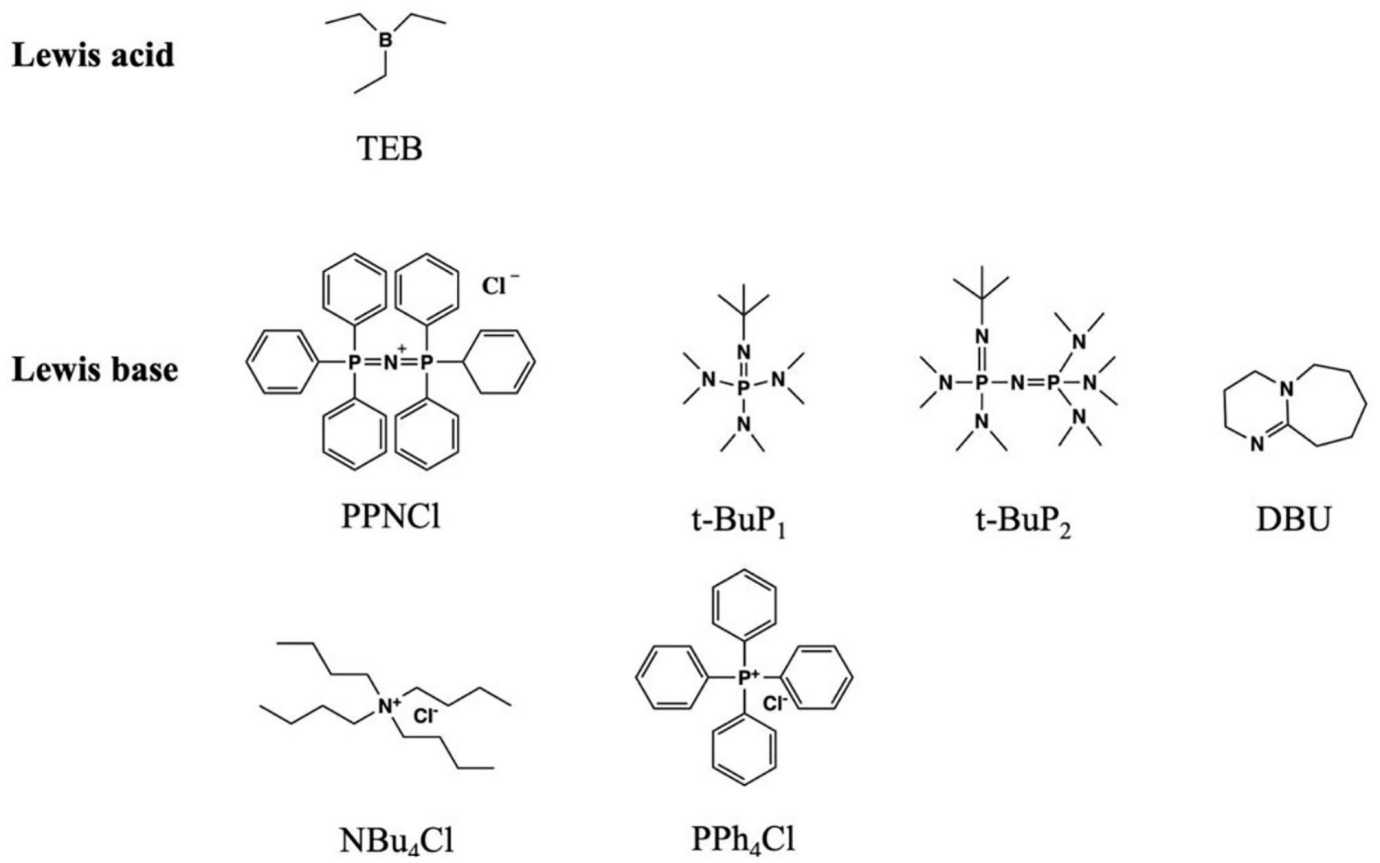
Lewis acid and Lewis base in this article.

The development of Lewis pair polymerization provides an efficient, controlled, and highly chemoselective approach to ROCOP. Compared with organometallic catalysts, the metal-free Lewis pair catalysts are easy to synthesize and often commercially available (Pappuru and Chakraborty, 2019; Hong, 2021). By adjusting the acidity, basicity, and steric effects, the Lewis acid–base pair can have excellent catalytic performance (Figure 8) (Zhang et al., 2020b). This section only summarizes the very recent highlights.

Chen et al. successfully used a Lewis pair consisting of TEB (as the Lewis acid) and [bis(triphenylphosphine) iminium chloride (PPNCl, as the Lewis base)] for the synthesis of alternating copolymers of CO_2_ and epoxides, including PGE and SO. By adjusting the TEB/PPNCl molar ratio, regioregular copolymers can be achieved. They proposed that TEB not only affects the balance of the TEB coordinating anions but also enhances the reaction activity of epoxides. Therefore, excess TEB can reduce the possibility of backbiting reaction. In addition, this study also investigated the catalytic ability of different Lewis pairs comprising TEB with a series of bases, such as tetrabutylammonium chloride (NBu_4_Cl), tetraphenylphosphonium chloride (PPh_4_Cl), 1,5-diazabicyclo[5.4.0]undec-5-ene (DBU), and a phosphazene base (*t*-BuP_2_), with the molar ratio of 6:1. It was found that NBu_4_Cl and PPh_4_Cl showed the lowest catalytic activities (TOFs of 12 and 13 h^−1^, respectively), where cyclic carbonate was the main product. Meanwhile, the DBU and *t*-BuP_2_ with TEB could not catalyze the coupling reaction. In terms of a temperature effect (50~80°C) on the ROCOP, it was found that a high reaction temperature would weaken the coordination structure, thus producing more free anions and promoting the backbiting reaction. In addition, the CO_2_/SO copolymers with moderate regional regularity could be obtained by using TEB/PPNCl as the catalyst system (Chen et al., 2019).

Similarly, in 2020, Ye et al. used TEB/PPNCl as catalysts to synthesize a tetrameric poly(ester-*b*-carbonate) copolymer using CO_2_, PO, PA, and CHO as monomers in a one-pot process. The block copolymer showed good biodegradability and a high *T*_g_ of >90°C. The introduction of CHO monomer provides rigidity and increases the *T*_g_ of the block copolymers. Adjusting monomer feeding ratios allows for controlling *T*_g_ values and tuning mechanical properties of the block copolymers. In addition, the block copolymers obtained in this study exhibit good transmittance (>85%) and high tensile strength (up to 54.8 MPa), which could be promising alternatives for conventional non-degradable polystyrene (Ye et al., 2020). Then, Zhang et al. used TEB/PPNCl as the catalysts to synthesize well-defined poly(ester-*b*-carbonate) with unimodal and narrow molecular weight distribution by one-pot and sequential approaches (Zhang et al., 2021).

**Figure 9:**
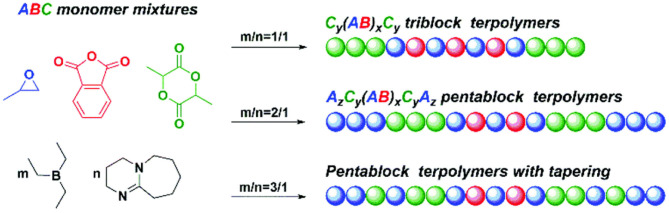
TEB/DBU pair catalyzed highly selective terpolymerization of mixtures of PO, PA, and *rac*-LA. TEB, triethyl borane; DBU, 1,8-diazabicyclo[5.4.0]undec-7-ene; PO, propylene oxide; PA, phthalic anhydride; LA, Lewis acid. Adapted with permission from Zhu et al. (2020). Copyright 2020 The Royal Society of Chemistry.

**Figure 10:**
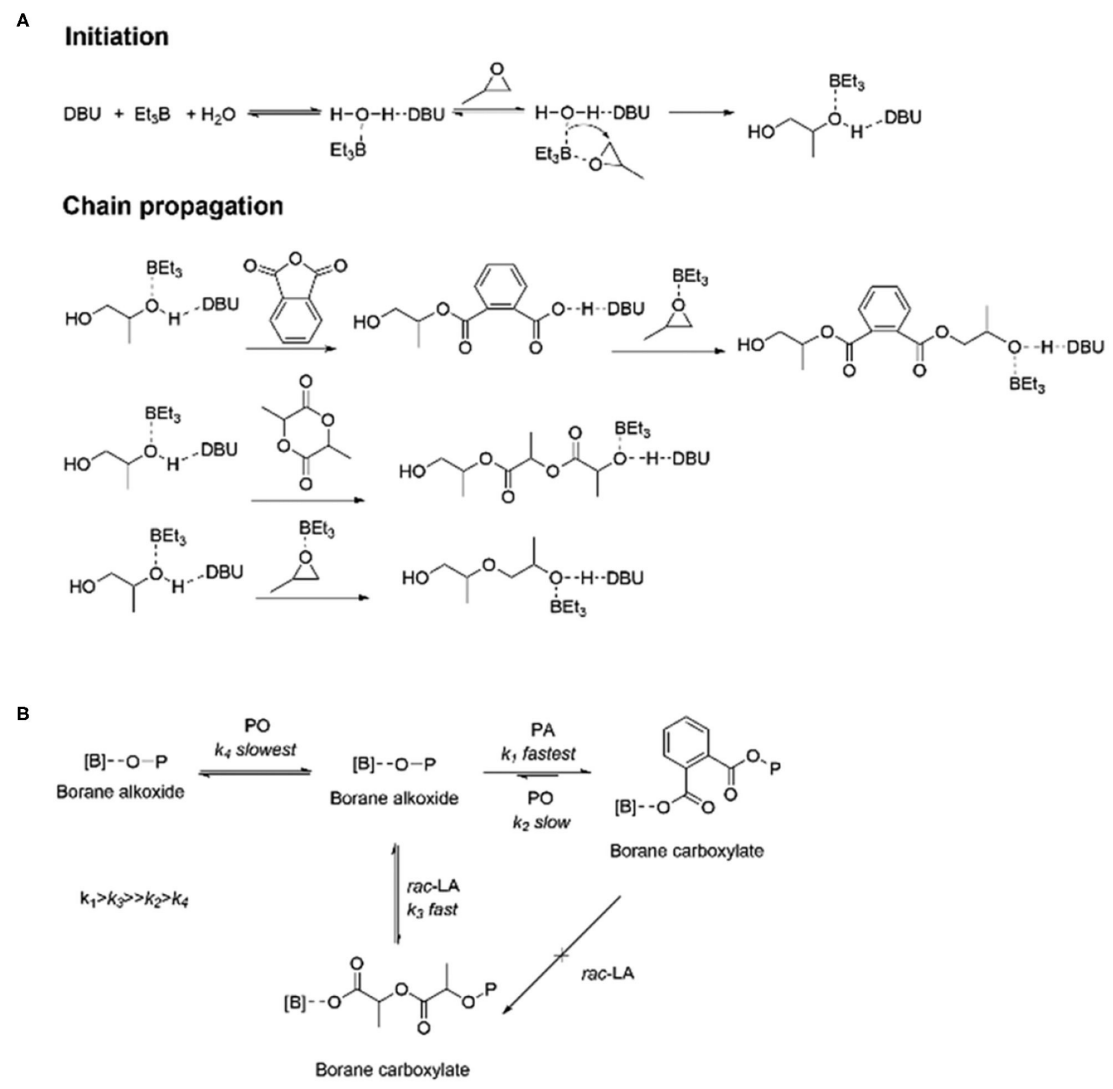
Plausible mechanistic aspects for the highly selective polymerization process: H_2_O-initiated ROCOP and ROP catalyzed by the TEB (Et_3_B)/DBU pair **(A)** and sequence controlled polymerization from monomer mixtures **(B)**. ROCOP, ring-opening copolymerization; ROP, ring-opening polymerization; TEB, triethyl borane; DBU, 1,8-diazabicyclo[5.4.0]undec-7-ene; LLA-OCA, l-lactide-*O*-carboxyanhydride. Adapted with permission from Zhu et al. (2020). Copyright 2020 The Royal Society of Chemistry.

In 2020, Zhu et al. used H_2_O as the initiator to catalyze the highly selective ROCOP of PO, PA, and *rac*-LA with TEB/DBU pair to synthesize α,ω-dihydroxy pentablock terpolymers in one pot (Figure 9; Zhu et al., 2020). First, separate catalytic experiments were carried out, and it was found that with TEB/DBU molar ratio being 1:1, the ROCOP of PO/PA showed a moderate catalytic activity and formed a completely alternating poly(trimethylene phthalate). Although the measured molecular weight was slightly lower than the theoretical value, the polymerization was still well-controlled, showing immortal polymerization features, even with the addition of H_2_O, and barely no decrease in the catalytic efficiency was observed. Interestingly, no PO-ROP was observed until the molar ratio of TEB/DBU increased to 2:1 and 3:1, indicating that additional TEB is needed to activate PO in the ROP of PO. Based on these results of separate polymerizations, the ROCOP of PO/PA/*rac*-LA was finally carried out in an autoclave with different TEB/DBU feed ratios using 1 equivalent of H_2_O as the initiator. At 80°C, the polymerization efficiency was high, and a near complete conversion (>99%) of PA and *rac*-LA was achieved within 5 h. During this terpolymerization, only until the complete consumption of PA was the ROP of *rac*-LA able to proceed. In addition, the presence of TEB might also prevent DBU from being an effective transesterification catalyst, so no transesterification occurred even during the extended reaction time. When the molar ratio TEB/DBU increased to 2:1 with 3 equivalent of H_2_O, the ROCOP of PO/PA, the ROP of *rac*-LA, and ROP of PO occurred sequentially at 60°C. However, when increasing the molar ratio to 3:1, PO started to homopolymerize even before the complete consumption of *rac*-LA, resulting in the formation of pentablock copolymers with tapering. This high chemoselectivity could be attributed to the orthogonal catalysis of the borane alkoxide intermediate, resulting from the ring opening of PO in the presence of water. It connects three polymerization cycles with different monomer insertion rates. The insertion rate of PA into the borane alcohol bond is much faster than that of *rac*-LA, while the insertion rate of *rac*-LA is faster than that of PO and highly affected by the molar ratio of TEB/DBU (Figure 10).

## Post-Polymerization Modification of Polymers Obtained by Ring-Opening Copolymerization

Functional groups such as hydroxyl groups or alkenes on polymer chains can be used as target sites for post-polymerization modification, which can further expand the application potentials of polymers (Farmer et al., 2018). Although the combination of epoxides and CO_2_/anhydrides can afford polycarbonates or polyesters by ROCOP, most of the obtained polycarbonates or polyesters lack functionality, restricting their high value-adding applications (Yang et al., 2018). In order to expand the application range of ROCOP polyesters and polycarbonates, epoxides, and anhydrides with different functional groups are of great value. Typical post-polymerization reactions are usually carried out by covalent modifications of the polymer end groups, main chain, and side groups (Gauthier et al., 2009). Among them, thiol-ene click chemistry is the most widely used method (Geschwind et al., 2013; Ntoukam et al., 2020). Besides, the formation of block copolymers or graft copolymers, bearing distinctive block properties (Ghosh et al., 2020; Gregory et al., 2020), could also be a facile approach toward functional materials, such as self-assembled nanomaterials. This section provides an overview of post-polymerization modification for functionalized polycarbonates and polyesters obtained by ROCOP in recent years.

**Figure 11:**
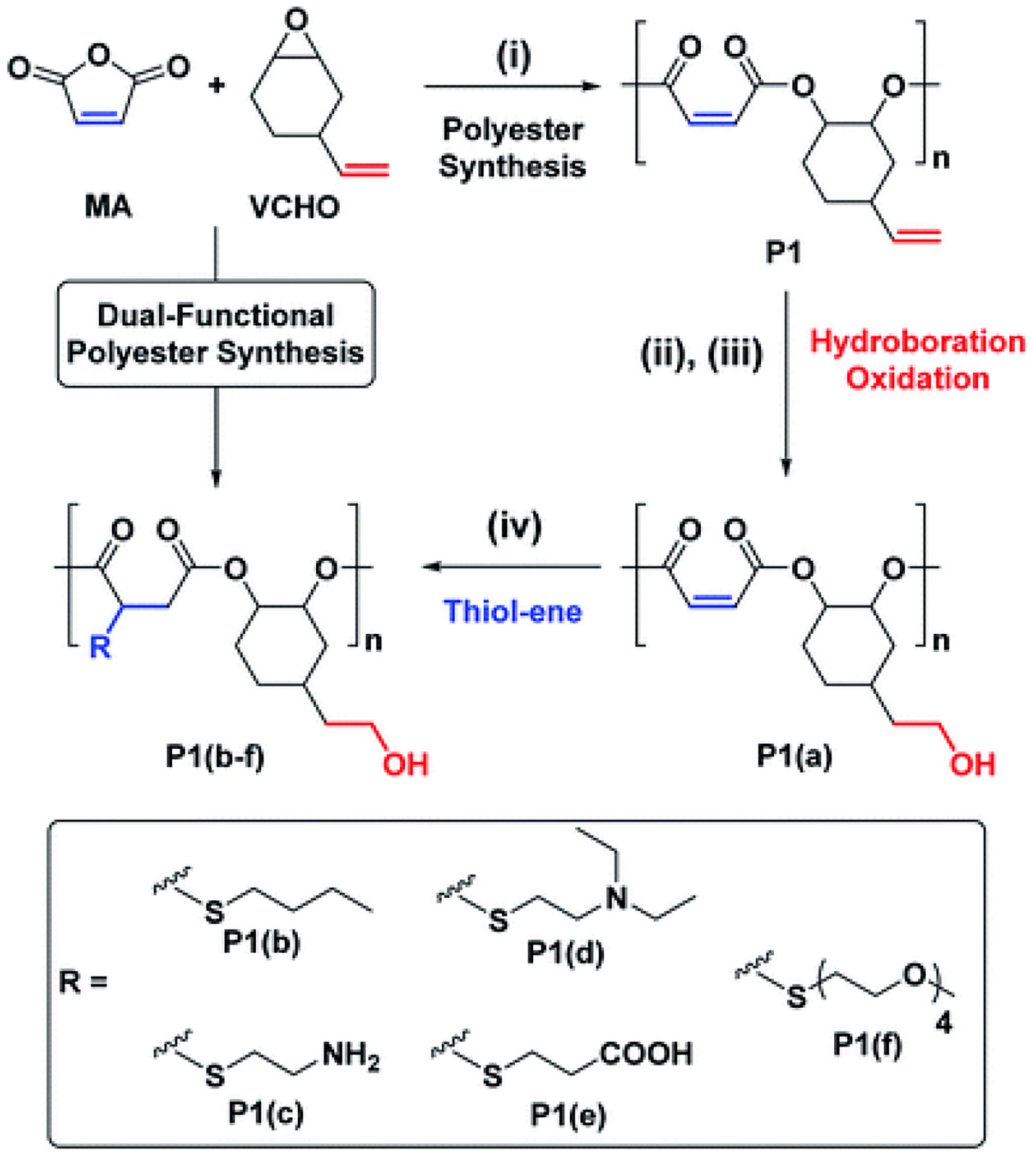
Selective synthesis of dual-functional alternating polyesters (using a combination of MA and VCHO as a representative example). Adapted with permission from Yi et al. (2019). Copyright 2019 The Royal Society of Chemistry.

Yi et al. synthesized highly alternating and orthonormal AB-type copolyesters with functional groups of terminal and internal alkenes using different alkene-containing epoxides [VCHO, vinyl PO (VPO), or allyl glycidyl ether (AGE)] and anhydrides [MA, tetrahydrophthalic anhydride (THPA), or tricyclic anhydride (TCA)], and SalcyCrCl/PPNCl as catalyst systems (Figure 11). The author first synthesized polyesters by ROCOP; after that, primary hydroxyl groups were introduced to the polyester chain *via* the selective hydroboration–oxidation reaction of the terminal alkenes. Then the internal alkenes were quantitatively converted into carboxylic acids, amines, alkyl, and oligo-groups groups by thiol-ene reactions. These copolymers with both hydrophobic and hydrophilic blocks can be self-assembled into nanostructures in aqueous solution, which is expected to be used as antimicrobial agents and biodegradable drug delivery vehicles (Yi et al., 2019).

**Figure 12:**
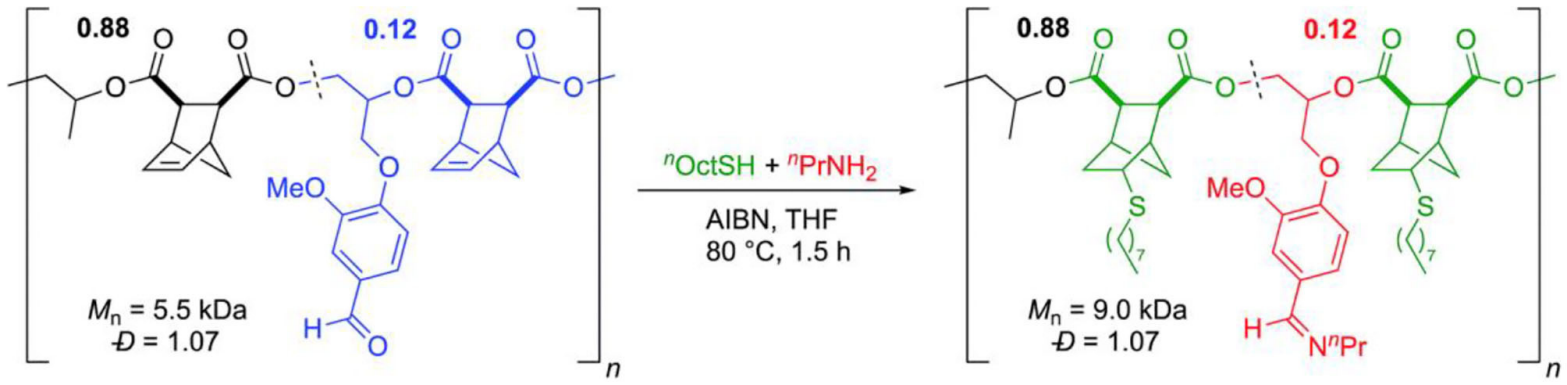
Orthogonal, one-pot, double post-polymerization modification reaction. Adapted with permission from Sanford et al. (2018). Copyright 2018 The Royal Society of Chemistry.

Sanford et al. used vanillin glycidyl ether (VGE) to directly introduce aldehyde functional group into the polymer chain by copolymerizing with PO and carbic anhydride (CPMA), using the [(^F^salph)Al(THF)][OTf]/[PPN]_2_[ADC] as a bifunctional catalytic system. Then, the poly(VGE-*alt*-CPMA) was treated by orthogonal post-polymerization modification (Figure 12). The aldehyde was reacted with an amine to form an imine linkage, which could be interesting in biomedical applications due to its pH sensitivity, while the alkene remained unreacted. Analogously, when the alkene was consumed completely through a thiol-ene reaction, the aldehyde remained intact. One step further, under the same reaction conditions, imine functionalization and thiol-ene reaction were combined into one system, achieving orthogonal and dual post-polymerization modification on aliphatic polyester in one pot (Sanford et al., 2018).

Chang et al. introduced tertiary amine functionalities into poly(PA-*alt*-VCHO) *via* the thiol-ene reaction. The wettability property of the obtained polymer showed that the introduction of tertiary amine groups in the polymer chain greatly improved the hydrophilicity of polymer nanofibers, leading to potential biomedical applications (Chang et al., 2018).

In 2014, Darensbourg and coworkers synthesized poly(cyclohexadiene carbonate) by ROCOP of CO_2_ with 1,2-epoxy-4-cyclohexene, and then they functionalized it by the thiol-ene reaction to afford amphiphilic polymer materials. Thereafter, the polymer was deprotonated with NH_4_OH aqueous solution to afford water-soluble polymers (Darensbourg et al., 2014). Shortly after, they also designed another amphiphilic block copolymer using the (salen)CoTFA/PPNTFA catalyst system. The hydrophobic block PPC was synthesized by the ROCOP of PO and CO_2_, and the PAGEC block was prepared by the ROCOP of AGE and CO_2_. The PAGEC block was then post-functionalized *via* the thiol-ene reaction to introduce carboxylic acids on the side chain, leading to the formation of amphiphilic triblock polycarbonates. The PAGEC-*b*-PPC-*b*-PAGEC can self-assemble into spherical nanoparticles (NPs) with a core-shell morphology in aqueous solution (Wang et al., 2015).

Besides thiol-ene chemistry, Diels–Alder reaction is another facile approach toward post-polymerization modification (Tasdelen, 2011). Hilf et al. prepared a polycarbonate, poly[(furfuryl glycidyl ether)-*co*-(glycidyl methyl ether)carbonate] poly[(FGE-*co*-GME)C], using furfuryl glycidyl ether (FGE), glycidyl methyl ether (GME) and CO_2_ as monomers, and (R, R)-(salen)-CoCl/PPNCl as the catalyst system (Hilf et al., 2015). The obtained polymer was then reacted with different maleimide derivatives [maleimide, *N*-(methoxycarbonyl) maleimide, and 1-(3,4-dihydroxyphenyl)-1*H*-pyrrol-2,5-dione(dopamine maleimide)] through Diels–Alder reaction to achieve reversible functionality.

## Potential Biomedical Applications of Functional Polyesters and Polycarbonates

Different from polyesters or polycarbonates prepared by ROP, ROCOP polycarbonates, or polyesters could potentially be more attractive for biomedical applications. As by simply changing the combination of epoxides and anhydrides, the material properties can be easily tuned. However, despite the significant progress made in catalytic systems, the biomedical potentials of the ROCOP polyesters and polycarbonates have not been well-studied, which is most likely due to the fact that ROCOP conditions are usually not compatible with certain functional groups, such as protic groups, and may induce inactivation/denaturation of the incorporated biomolecules (Feng et al., 2012; Dai and Zhang, 2019). Moreover, the organometallic residuals could be cytotoxic, and the degradation behavior of polymers prepared by ROCOP still lacks proper investigation (Xu et al., 2020). In this section, a few pioneering studies of ROCOP polymers for potential biomedical applications will be outlined.

Integrating bio-imaging and drug delivery into one system makes it possible to combine diagnosis and treatment for diseases such as cancer (Wang et al., 2018). Li et al. developed a reliable and effective tumor imaging platform with ROCOP polycarbonates (Li et al., 2017b). The triblock polycarbonates, poly(allyl glycidyl ether carbonate)-*b*-poly(propylene carbonate)-*b*-poly(allyl glycidyl ether carbonate) (PAGEC-*b*-PPC-*b*-PAGEC), was prepared by sequential epoxide addition during the ROCOP. The obtained triblock polycarbonates were then modified by the thiol-ene reaction between the allyl groups on the PAGEC block and Boc-protected cysteine. Amphiphilicity was achieved after the deprotection of Boc. The amphiphilic triblock polycarbonates were labeled with gadolinium (Gd^3+^) and self-assembled into polymeric micelles, which showed relatively low cytotoxicity both *in vitro* and *in vivo*. The polymer micelles were degradable *via* the hydrolysis of carbonate bonds to release DPTA/Gd complexes. The tumor MRI showed that these Gd-loaded polymer micelles have excellent MR imaging ability. This study demonstrated that CO_2_-based amphiphilic polycarbonates with improved hydrophilicity and functionality can be used as an efficient platform for tumor imaging (Li et al., 2017b).

**Figure 13:**
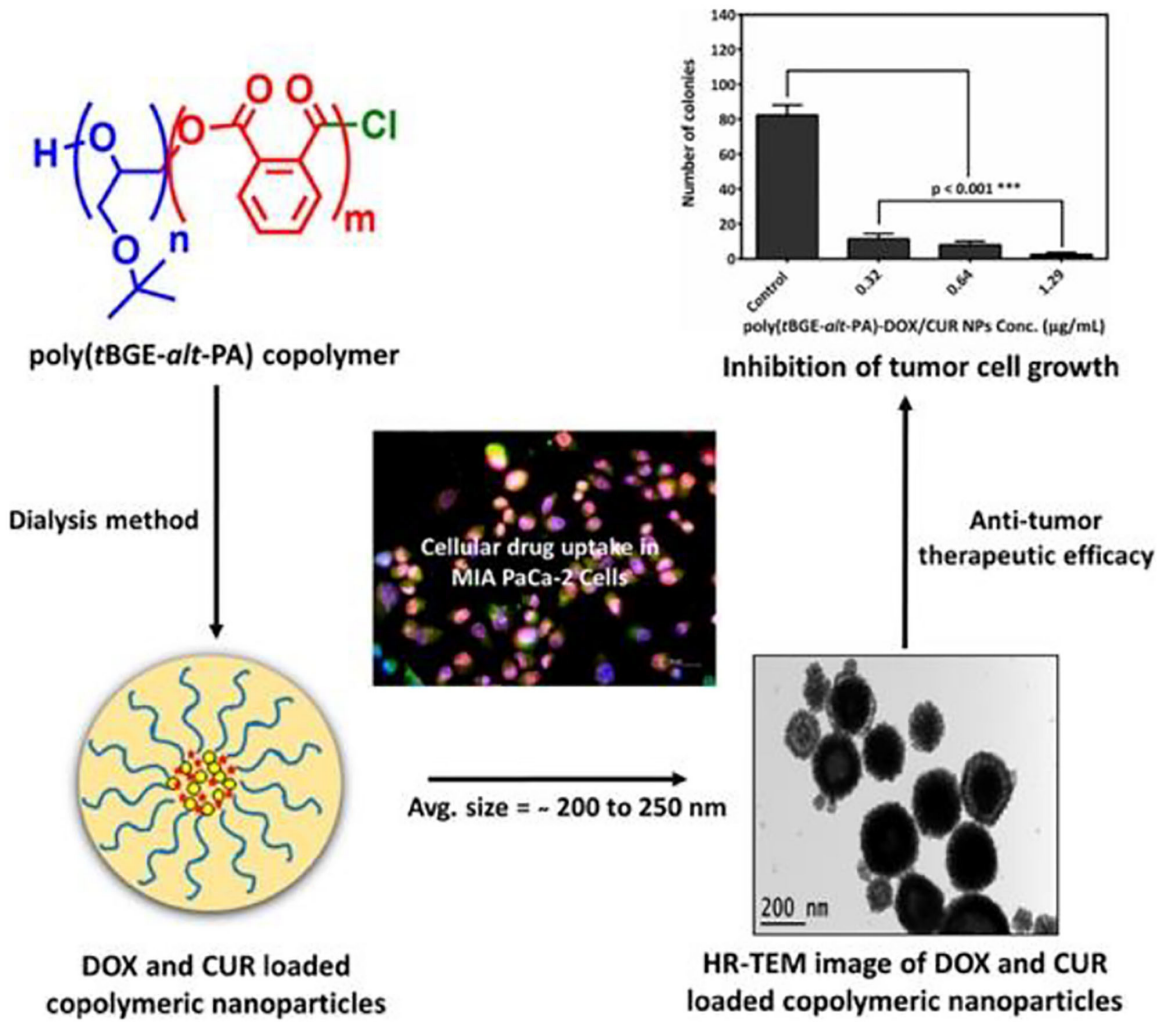
Illustration of doxorubicin (DOX) and curcumin (CUR) loaded nanoparticles self-assembled from poly(*t*BGE-*alt*-PA). Adapted with permission from Gupta et al. (2019). Copyright 2019 Elsevier.

Doxorubicin (DOX) and curcumin (CUR) have an anti-tumor activity; some studies have shown that CUR expresses its chemo-sensitization action by increasing its accumulation in cancer cells to enhance the anti-tumor ability of DOX. Based on this, Gupta et al. prepared different drug-loaded NPs, consisting of poly(*t*BGE-*alt*-PA) with DOX and CUR, and the poly(*t*BGE-*alt*-PA) was synthesized with *tert*-butyl glycidyl ether (*t*BGE) and PA using the TEB/PPNCl catalyst system (Figure 13). Although poly(*t*BGE-*alt*-PA) is biodegradable, TGA results suggested that NPs can be stored for a long time at room temperature. Drug release experiments showed that the *in vitro* drug release of the NPs followed anomalous transport pattern, with higher cellular drug uptake of DOX and CUR in the NP formulation, compared with free drug. Moreover, drug-loaded NPs had no significant cytotoxicity against MCF-10A cells, and the dual-drug system also showed higher anticancer therapeutic efficacy (Gupta et al., 2019). Kummari et al. tested a few metal-free Lewis pairs on the alternating copolymerization of norbornene anhydride and different epoxides. The obtained polyesters showed good biocompatibility upon human embryonic kidney cells (HEK-293) (Kummari et al., 2019).

**Figure 14:**
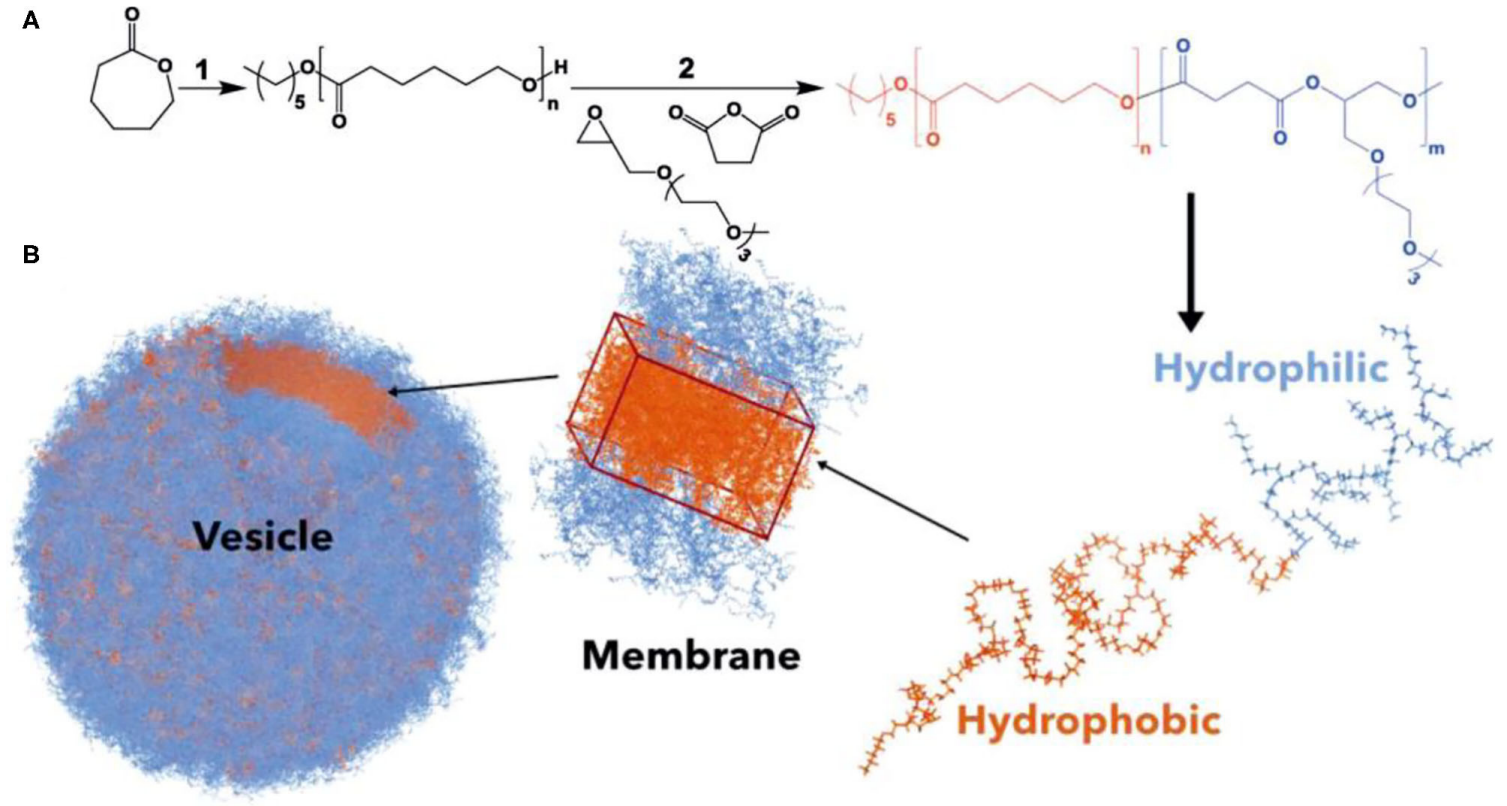
**(A)** Synthesis of PCL-*b*-PE. **(B)** PCL-*b*-PE forms polymersomes *via* self-assembly. Adapted with permission from Zhu et al. (2019). Copyright 2019 Wiley.

Zhu et al. demonstrated the significance of polymer degradation products, besides being used simply as delivery vehicles, in metabolic activities to modulate cell behavior. They used PCL as the macroinitiator to initiate the ROCOP of succinic anhydride (SA) and 2-[(2-(2-(2-meth-oxyethoxy)ethoxy)ethoxy)methyl]oxirane (ME_3_MO), leading to the formation of amphiphilic PCL-*b*-PE block copolyesters (Figure 14). These polymers formed vesicle-like nanostructures via self-assembly. The self-assembled nanostructure was able to be degraded rapidly by lipase to afford metabolites including succinic acid, 6-hydroxyhexanoic acid, and a derivative of glycerol containing tri(ethylene glycol) substituent. These degradation products were found to stimulate the proliferation of dermal fibroblasts, which could be of great value for cell-specific wound healing. This finding signals the importance of designing drug delivery systems with specific metabolic activities (Zhu et al., 2019).

**Figure 15:**
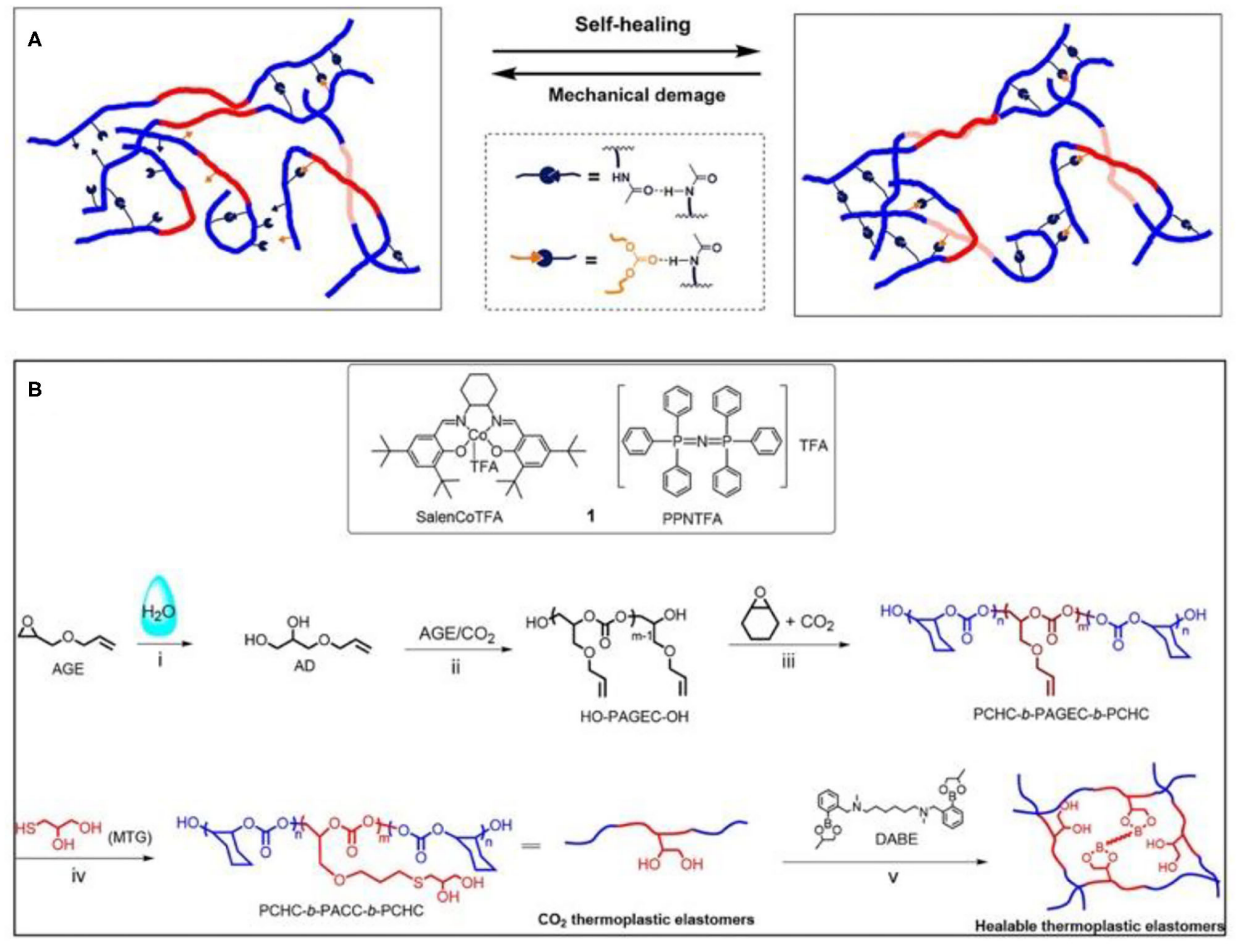
**(A)** Schematic illustration of the self-healing mechanism of the CO_2_-based block copolymers (Yang et al., 2018). Adapted with permission from Yang et al. (2018). Copyright 2018 American Chemical Society. **(B)** SalenCoTFA/PPNTFA catalyst system and tandem strategy performed for the preparation of CO_2_-based healable thermoplastic elastomers (Yang and Wu, 2019). Adapted with permission from Yang and Wu (2019). Copyright 2019 American Chemical Society.

Inspired by the self-healing ability of living organisms, synthetic biomedical self-healing materials have also been extensively studied. Self-healing materials can automatically repair damage to return their original shape, structure, functionality, and physical and chemical properties without external intervention (Chen et al., 2017; Shi et al., 2019). At the molecular level, self-healing methods can be divided into physical events and chemical events (for instance, covalent bonding, free radicals, and supramolecular dynamic bonds; Wang and Urban, 2020). At present, in order to make self-healing materials with good mechanical properties and self-healing ability at the same time, the commonly used method is to prepare multi-block copolymers, in which the hard block provides strength and rigidity for the material and the multivalent supermolecular interactions ensure self-healing (Chen and Guan, 2014; Lee et al., 2020). Darensbourg and Wu's group synthesized a series of CO_2_-based polycarbonate elastomers with self-healing ability *via* a one-pot method, combining ROCOP with the thiol-ene reaction as the post-polymerization modification strategy. Triblock copolymer, PAGEC-*b*-PPC-*b*-PAGEC, was prepared first ROCOP. Then, excess CO_2_ was removed after the consumption of AGE, followed by the addition of 2-amido ethanethiol to perform thiol-ene modification (Figure 15A), introducing self-healing ability (Yang et al., 2018). Due to the reversible hydrogen bonding effect, the obtained polycarbonates showed good self-healing ability and can almost completely recover without any external stimulation at room temperature. Besides, almost no loss of the mechanical properties and self-healing ability were observed even after repetitive thermoforming (Yang et al., 2018). Another healable CO_2_-based thermoplastic elastomer, PCHC-*b*-PACC-*b*-PCHC, was synthesized *via* the addition of *N*1,*N*6-dimethyl-*N*1,*N*6-bis(2-(4-methyl-1,3,2-dioxaborolan-2-yl)benzyl)-hexane-1,6-diamine (DABE) as a dynamic cross-linker (Figure 15B). Monothioglycerol was added to perform the thiol-ene click reaction. The experimental results showed that before modification, the block polymer had only one *T*_g_ (~20°C), but after modification, two *T*_g_ values (~−20 and ~100°C) appeared, suggesting the formation of microphase separation and increased hydrophilicity of PACC blocks. The PCHC block offers rigidity, while the dynamic exchange of boronic ester bond ester between the diol groups and telechelic diboronic esters in the soft PACC block/DABE phase contributes self-healing (Yang and Wu, 2019).

## Conclusion and Outlook

Compared with other controlled polymerization reactions, ROCOP provides a more facile method to tailor the material properties of polyesters and polycarbonates by simply changing the monomer combinations of epoxides, CO_2_, and cyclic anhydrides. The catalysis of ROCOP has been investigated for decades, and a few comprehensive reviews have been reported. However, the recent development in multi-nuclear organometallic catalytic systems and metal-free catalytic systems has rarely been summarized. Multi-nuclear organometallic catalysts, especially the heteronuclear ones, generally exhibited higher polymerization rates and polymer selectivities than those of the mononuclear analogs, while the metal-free catalytic system is of great potential to be used for the synthesis of biopolymers. Nevertheless, there are still a few main issues to be addressed, including the lack of understanding of elementary steps during the ROCOP, rigorous polymerization conditions (e.g., high CO_2_ pressure and high reaction temperature), the lack of stereoselective catalytic systems, and the less well-controlled monomer/block sequence.

In terms of the material properties, though protonic functional groups may not be tolerated during the ROCOP, there are great potentials to introduce functionalities *via* the post-polymerization modification. The most commonly used approaches are the thiol-ene reaction, hydroboration–oxidation reaction, and Diels–Alder reaction. Most importantly, owing to the alternating monomer sequence in ROCOP polymers, orthogonal functionality is easily accessible to reach versatile properties. Such versatility would be a great advantage for biomedical applications, where no single property fits all needs. However, compared with the vast advances in catalysis study, the potential bioapplications of ROCOP polymer have not attracted enough attention. This leaves many opportunities for the future development of ROCOP polymers tailored for biomedicine, including the accurate control of degradation rate to accommodate different application scenarios, understanding the metabolic activity of degradation products, and introducing versatile functionalities with minimum induced toxicity.

## Author Contributions

All authors listed have made a substantial, direct and intellectual contribution to the work, and approved it for publication.

## Conflict of Interest

The authors declare that the research was conducted in the absence of any commercial or financial relationships that could be construed as a potential conflict of interest.

## Abbreviations

AGE: allyl glycidyl ether
BA: benzoate
*t*-BGE: *tert*-butyl glycidyl ether
BilBTP: bis(benzotriazolyliminophenolate)
BTA: butyrate
*t*-BuP_1_: *tert*-butyliminotris(dimethylamino)phosphorane
CBO, *cis*-2: 3-epoxybutane
*trans*-2, 3-CBO, *trans*-2: 3-butylene oxide
CDO, 1: 4-dihydro-naphthalene oxide
CEO, 1: 2-epoxy-4-cyclohexene
CHC: cyclohexene carbonate
CHO: cyclohexene oxide
Cl-SO: 4-chlorostyrene oxide
CO_2_: carbon dioxide
COPO, 3: 4-epoxytetrahydrofuran
CPMA: carbic anhydride
CPO: cyclopentene oxide
*trans*-CSO: *trans*-stilbene oxide
(*S, S*)-CSO, *rac*-/(*S*: *S*)-*trans*-diethyl epoxysuccinate
*rac*-*trans*-CSO: *rac*-*trans*-stilbene oxide
*trans*-CTO, *rac*-/(*S*: *S*)-*trans*-diethyl epoxysuccinate
CTPB: cyclic trimeric phosphazene base
DBU, 1: 8-diazabicyclo[5.4.0]undec-7-ene
DFT: density functional theory
DHC: dihydrocoumarin
DIBTP: diamine-bis(benzotriazole phenolate)
DGA: diglycolic anhydride
ε-DL: ε-decalactone
DMAP: 4-dimethylaminopyridine
3, 4-DMPA, 3: 4-dimethyl phthalic anhydride
DPA: diphenic anhydride
DSC: differential scanning calorimetry
EC: ethylene carbonate
ECH: epichlorohydrin
GA: glutaric anhydride
GO: phenyl glycidyl ether
HA: hydrocinnamate
LA: Lewis acid
*rac*-LA: *rac*-lactide
LB: Lewis base
LLA-OCA: l-lactide-*O*-carboxyanhydride
MA: maleic anhydride
MEA: monoethanolamine
NA, *cis*-5-norbornene-*endo*-2: 3-dicarboxylic anhydride
1, 2-NA: 2-naphthalic anhydrides
PA: phthalic anhydride
PCHC: poly(cyclohexene carbonate)
PCHPE: poly(cyclohexylene phthalate)
PEC: poly(ethylene carbonate)
PLA: poly lactide
PLLA-*b*-PCHC: poly(l-lactide-*b*-cyclohexene carbonate)
PO: propylene oxide
PPNCl: bis(triphenylphosphine)iminium chloride
ROP: ring-opening polymerization
ROCOP: ring-opening copolymerization
SA: succinic anhydride
SO: styrene oxide
TCA: tricyclic anhydride
TEB: triethyl borane
TFPB: tris(pentafluorophenyl)borane
THPA: tetrahydrophthalic anhydride
TPB: triphenyl borane
TON: turnover number
TOF: turnover frequency
VCHO: vinyl cyclohexene oxide
VGE: vanillin glycidyl ether
VPO: vinyl propylene oxide.
